# De novo Phased Genome Assembly, Annotation and Population Genotyping of *Alectoris Chukar*

**DOI:** 10.1038/s41597-024-02991-0

**Published:** 2024-02-02

**Authors:** Hao Zhou, Xunhe Huang, Jiajia Liu, Jinmei Ding, Ke Xu, Wenqi Zhu, Chuan He, Lingyu Yang, Jianshen Zhu, Chengxiao Han, Chao Qin, Huaixi Luo, Kangchun Chen, Shengyao Jiang, Yurou Shi, Jinyuan Zeng, Zhuoxian Weng, Yongjie Xu, Qing Wang, Ming Zhong, Bingwang Du, Sen Song, He Meng

**Affiliations:** 1 Shanghai Collaborative Innovation Center of Agri-Seeds/School of Agriculture and Biology, Shanghai Jiao Tong University, Shanghai, 200240 China; 2 Jiaying University/Guangdong Provincial Key Laboratory of Conservation and Precision Utilization of Characteristic Agricultural Resources in Mountainous Areas, Meizhou, 514015 China; 3 School of Life Sciences, Lanzhou University, Lanzhou, 730000 China; 4 Department of Animal Science, Guangdong Ocean University, Huguangyan East, Zhanjiang, Guangdong 524088 China

**Keywords:** Comparative genomics, Agricultural genetics, Evolutionary genetics, DNA sequencing

## Abstract

The *Alectoris Chukar* (chukar) is the most geographically widespread partridge species in the world, demonstrating exceptional adaptability to diverse ecological environments. However, the scarcity of genetic resources for chukar has hindered research into its adaptive evolution and molecular breeding. In this study, we have sequenced and assembled a high-quality, phased chukar genome that consists of 31 pairs of relatively complete diploid chromosomes. Our BUSCO analysis reported a high completeness score of 96.8% and 96.5%, with respect to universal single-copy orthologs and a low duplication rate (0.3% and 0.5%) for two assemblies. Through resequencing and population genomic analyses of six subspecies, we have curated invaluable genotype data that underscores the adaptive evolution of chukar in response to both arid and high-altitude environments. These data will significantly contribute to research on how chukars adaptively evolve to cope with desertification and alpine climates.

## Background & Summary

*Alectoris Chukar* (chukar) commonly known as “chukar”, is a member of the Galliformes order and the Phasianidae family, hailing from the stony semi-desert regions of Asia, Western Europe, and the Middle East. This species has been introduced to numerous other countries such as the United States, Canada, England, and New Zealand, primarily for stocking on game farms or releasing for hunting purposes^1,2^. In recent years, there has been an uptick in the use of chukars for meat production under controlled husbandry conditions. Owing to their rapid growth, high productivity, and superior meat quality, chukars are ideally suited for commercial production^3–6^. The domestication of these partridges coupled with selection for growth traits enhances their potential as a prime source of high-quality protein for human consumption.

The wild chukar, a polytypic species with 22 subspecies scattered globally, exhibits a broad spectrum of environmental adaptations. Among these are the six subspecies of pubescens, potanini, pallida, falki, dzungarica, and pallescens, with pubescens and pallida exclusively found in China. These subspecies have evolved to survive in their specific habitats, which span a wide range of temperatures and altitudes. For instance, while falki is adapted to a drier environment, pallescens thrives in the highlands of Tibet (altitude >4000 m).

This environmental adaptation capacity and genetic diversity among the chukar subspecies underline the significance of comprehensive genomic research in this species. Whole-genome sequence assembly has proven to be a fundamental tool for extensive genomics initiatives, including evolutionary studies and efficient breeding strategies. Over the years, critical poultry species like chickens, turkeys, and ducks have substantially reaped the benefits of these genomic resources^7–10^. Notably, the chicken reference genome has undergone several refinements, making it one of the superior vertebrate genomes available and establishing it as a model for avian research^9^. However, the chukar partridge’s genomic advancement is currently stymied due to the absence of a reference genome. The introduction of phased-genome assembly, renowned for its precision in resolving complex genomic variations^11^, could be instrumental in breaking this impasse, thus transforming the genetic improvement of chukar breeding, spurring evolutionary studies, and securing genetic resource conservation.

In this study, we employ a *de novo* assembly strategy to present the first continuous, accurate, phased-resolved genome for the chukar. Utilizing this genome, we resequenced and analyzed five wild subspecies and one domestic population of chukar. Our research provides valuable resources for investigating adaptive evolution, breeding, and conservation genomics of crucial ecological species.

## Method

### Ethics statement

The collection and handling of the samples in this study were carried out in accordance with approved guidelines and regulations from both Lanzhou University and Shanghai Jiao Tong University.

### Sample collections

**Fig. 1:**
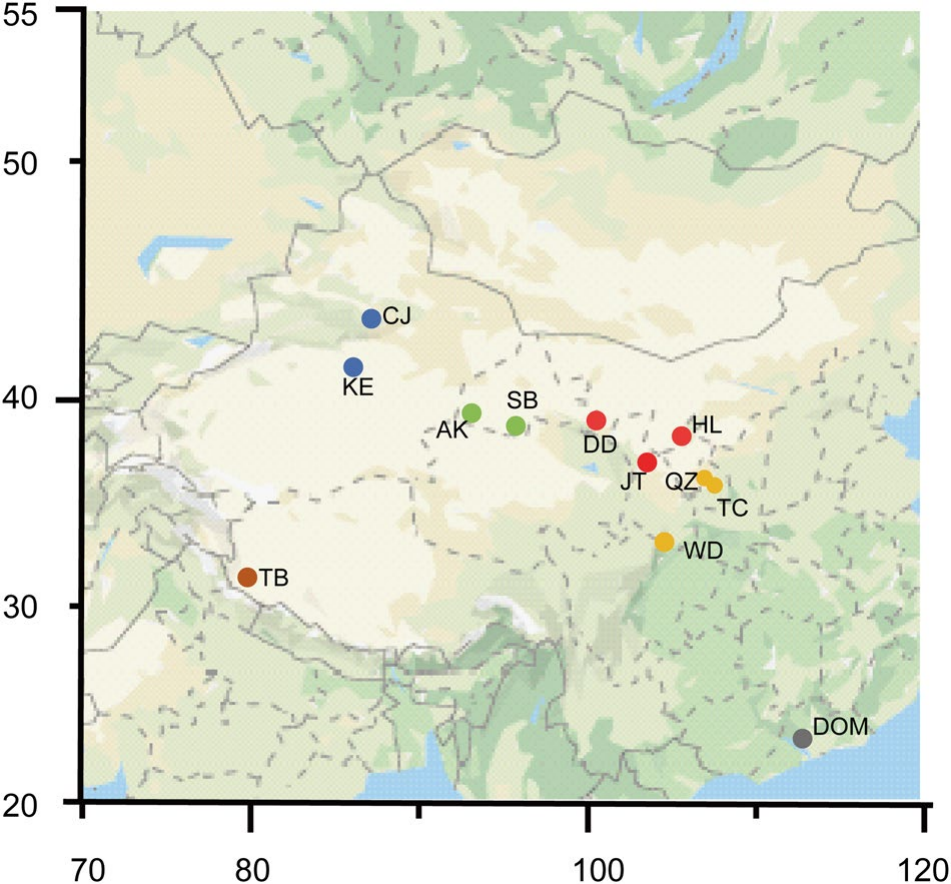
Sampling distribution map of chukar subspecies. Pubescens (orange), potanini (red), pallida (green), falki (blue), pallescens (brown), and domestic (pink) samples were obtained from 12 distinct locales including Akesai(AK), Changji(CJ), Dongdashan(DD), Helanshan(HL), Jingtai(JT), Kuerle(KE), Quzi (QZ), Subei (SB), Tongchuan (TC),Wudu (WD), Tibet (TB), and Jieyang(DOM). The horizontal axis represents longitude, and the vertical axis represents latitude.

We sequenced the genome of a female domesticated chukar collected from Tianming *Alectoris chukar* Farm (Guangzhou, Guangdong, China), which was primarily used for genome assembly. The transcriptomes of two adult female domesticated chukars, which were collected from Tianming *Alectoris Chukar* Farm (including the one used for genome assembly), were sequenced for annotation of coding genes in the genome. In addition, we included the genomes resequenced from a total of 58 chukars for genotype identification. These samples include 14 pubescens, 12 potanini, 9 pallida, 14 falki, 2 pallescens, and 7 domestic chukars (refer to Table 1 for more details). Pubescens, potanini, pallida, and falki muscle samples were obtained from 10 distinct locales including Akesai(AK), Changji(CJ), Dongdashan(DD), Helanshan(HL), Jingtai(JT), Kuerle(KE), Quzi (QZ), Subei (SB), Tongchuan (TC) and Wudu (WD) during 2002–2008, representing the majority of chukar’s geographical area in China and reflecting various geographic, topographic, and climatic conditions (Fig. 1, Table 1). To avoid sampling near relatives, each bird within a location was collected from a different portion of the colony. These 49 samples were donated by Lanzhou University. Three and four blood samples were collected at random from domestic (DOM) chukars in Tianming farm (Jieyang, China) and Qinxiangyuan farm (Jieyang, China), respectively. To avoid the selection of relatives, the pedigrees of these DOM chukar were investigated (Table 1). Two muscle samples of pallescens sampled in Tibet (TB) province were received from the animal branch of the southwest China germplasm bank of wildlife (Yunnan, China) (Table 1).

**Table 1:** Sample sizes and sampling locations of chukar subspecies.

Subspecies	Latitude	Longitude	Altitude(m)	Location	Province	Abbreviations	Sample size
pubescens	33°23′33″	104°55′34″	1005	Wudu	Gansu	WD	5
	36°00′56″	107°30′38″	1455	Tongchuan	Gansu	TC	4
	36°26′06″	107°20′40″	1450	Quzi	Gansu	QZ	5
potanini	38°34′11″	105°57′12″	1366	Helanshan	Ningxia	HL	4
	37°09′11″	103°54′11″	1893	Jingtai	Gansu	JT	4
	39°04′38″	100°48′06″	2798	Dongdashan	Gansu	DD	4
pallida	39°30′45″	94°52′37″	2287	Subei	Gansu	SB	4
	39°22′32″	94°14′56″	3050	Akesai	Gansu	AK	5
falki	41°47′33″	86°0937″	1065	Kuerle	Xinjiang	KE	8
	43°59′10″	87°14′39″	624	Changji	Xinjiang	CJ	6
pallescens	31.48345	79.80255	4000	Ali	Tibet	TB	2
domesticatic	116°21′21″	23°32′38″	20	Jieyang	Guangdong	DOM	3
	116°21′21″	23°32′38″	20	Jieyang	Guangdong	DOM	4

### ***De novo*** sequencing and assembly of the chukar phased genome

The DNA samples of the female domesticated chukar blood were extracted and sequenced using PacBio single-molecule real-time (SMRT) sequencing and Illumina paired-end sequencing technology. We carried out SMRT DNA sequencing of ∼20 kb inserts using the PacBio Sequel II platform (Personal Biotechnology Co., Ltd. Shanghai, China). Next, 400 bp paired-end libraries (refer to Illumina TruSeq DNA Sample Preparation Guide) constructed from the same genomic DNA were sequenced on the Illumina HiSeq platform (Personal Biotechnology Co., Ltd. Shanghai, China). The DNA sample of the chukar muscle was extracted and sequenced using Hi-C technology in Beijing Nuohezhiyuan Technology Service Co, Ltd. We filtered and trimmed the Illumina and Hi-C reads to remove adapters and low-quality bases using the standard settings in SOAPnuke v1.5.0^12^.

After low-quality and adaptor reads were filtered, we obtained ~136.59 Gb long sequencing data that were used to assemble the genome (Table S1a). *De novo* assembly followed the PacBio string graph assembler process, using FALCON v2.1.4, FALCON-Unzip v0.4.0, and FALCON-phase v0.2.0^13^ to generate long-range phased haplotypes. FALCON computes an initial assembly by correcting errors in raw reads and subsequently assembling them using a string graph formulated from read overlaps. Following this, FALCON-Unzip identified read haplotypes based on the phasing information derived from detected heterozygous positions. These phased reads are then deployed to assemble both haplotigs and primary contigs. As a result of this assembly process, there were 363 and 1,711 contigs for the primary contigs and haplotigs respectively (Table 2). The cumulative lengths of these two haploid assemblies were 1.03 Gb and 0.85 Gb, with their Contig N50 lengths reaching approximately 29.3 Mb and 1.1 Mb each. Subsequently, FALCON-Phase inputs the partially phased long-read assembly from FALCON-Unzip, and extends the phasing on the contigs using ~182 Gb filtered Hi-C data from the same sample. This achieves a phased contig-level genome assembly of chukar, resulting in the production of both Hap1 Contigs and Hap2 Contigs with a total length of 1.03 Gb and contig N50 length of 1.06 Mb (Table 2, Table S1a). Furthermore, Jellyfish v2.1.4 (https://github.com/gmarcais/Jellyfish) was used in conjunction with GenomeScope v1.0.0^14^ to calculate genome size and heterozygosity in the chukar genome a k-mer frequency of 18. The genome size estimated (~1.0 Gb) was consistent with the genome assembled (Fig. 4a).

**Table 2:** Summary of genome assembly of Alectoris Chukar.

	Pirmary Contigs	Haplotigs	Hap1 Contigs	Hap2 Contigs	Hap1 Scaffolds	Hap2 Scaffolds
Record number	363	1,711	3,188	3,188	186	183
Sum of length	1,033,291,773	856,644,580	1,034,640,777	1,033,372,611	1,034,932,086	1,033,664,093
Average length	2,846,533	500,668	324,542	324,144	5,564,151	5,648,437
Longest length	113,559,260	5,264,089	5,264,089	5,251,291	199,027,886	198,928,213
Count (>1 kb)	363	1,710	3,056	3,056	181	179
Count (>60 kb)	235	1,535	1,983	1,978	51	51
N20	55,683,641	2,227,792	2,221,606	2,222,945	151,151,867	151,023,354
N50	29,306,462	1,125,139	1,069,603	1,069,603	93,605,039	93,464,300
N90	4,633,874	205,917	152,474	153,014	15,133,858	12,983,908
Count (N20)	3	55	66	66	2	2
Count (N50)	10	222	275	275	4	4
Count (N90)	37	853	1,219	1,215	16	17

**Fig. 2:**
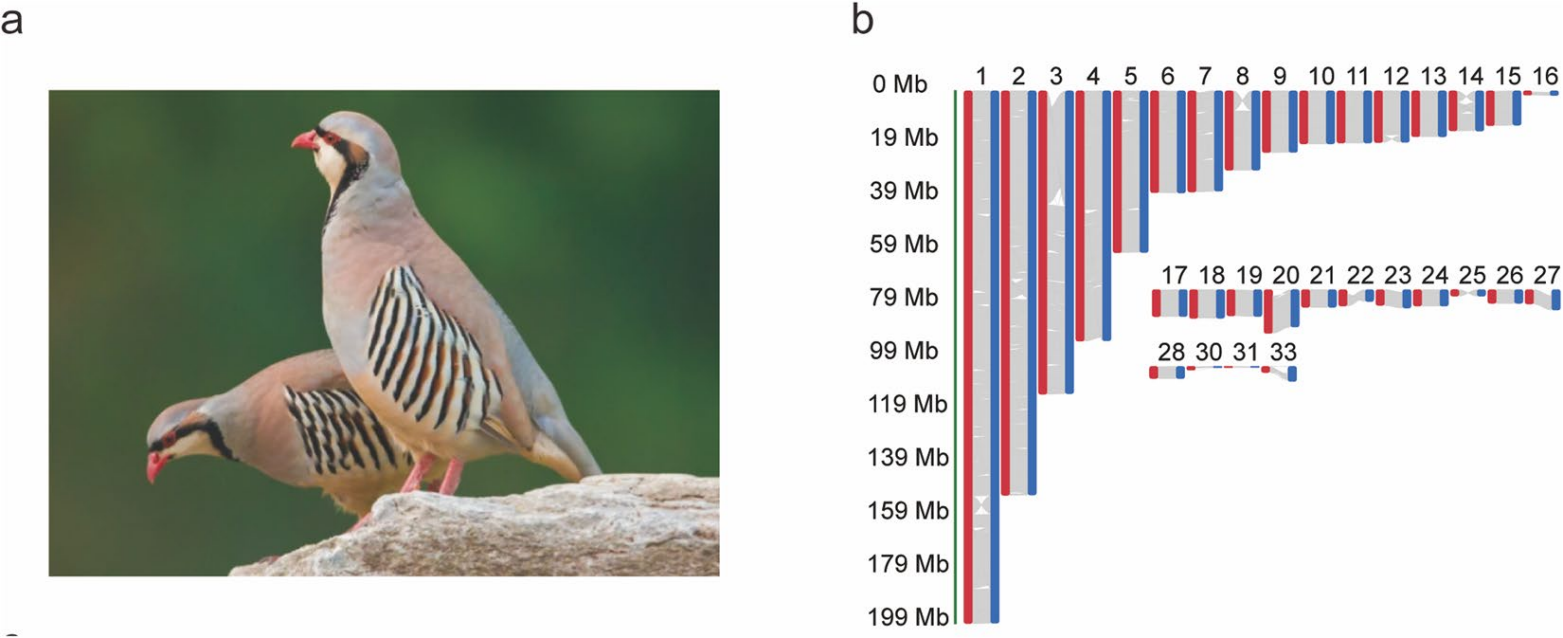
Diagram of genome assembly and phylogenetic relationship of *Alectoris chukar* (chukar). (**a**) Photograph of the chukar. (**b**) Synteny map of primary and associated assembly.

After the contig-level assemblies were generated, they were respectively polished using Pilon v1.22^15^, with the help of ~124.12 Gb high-quality Illumina data. Scaffolding was performed on these polished sequences using ALLHiC v0.9.8^16^, with the support of ~182 Gb Hi-C sequencing data. In our next steps, Ragag Scaffold v1.1.0^17^ was employed for a reference genome-assisted methodology to construct more comprehensive chukar haplotype genomes and ascertain chromosome IDs of scaffolds. The chicken chromosome-assembled genome (GRCg6a)^18^ served as the reference due to its close evolutionary relationship with chukar and shared chromosome number (n = 78)^19^. This stage merely oriented and ordered draft assembly sequences into longer sequences without modifying the input query sequence. Finally, we generated two phased pseudo-haplotype genomes of chukar (Hap1 Scaffolds and Hap2 Scaffolds) with 31 pairs of chromosomes (Fig. 2b and Table S2). The N50 length for both Hap1 Scaffolds and Hap2 Scaffolds was significantly enhanced, reaching 93.6 Mb and 93.5 Mb respectively.

**Fig. 3:**
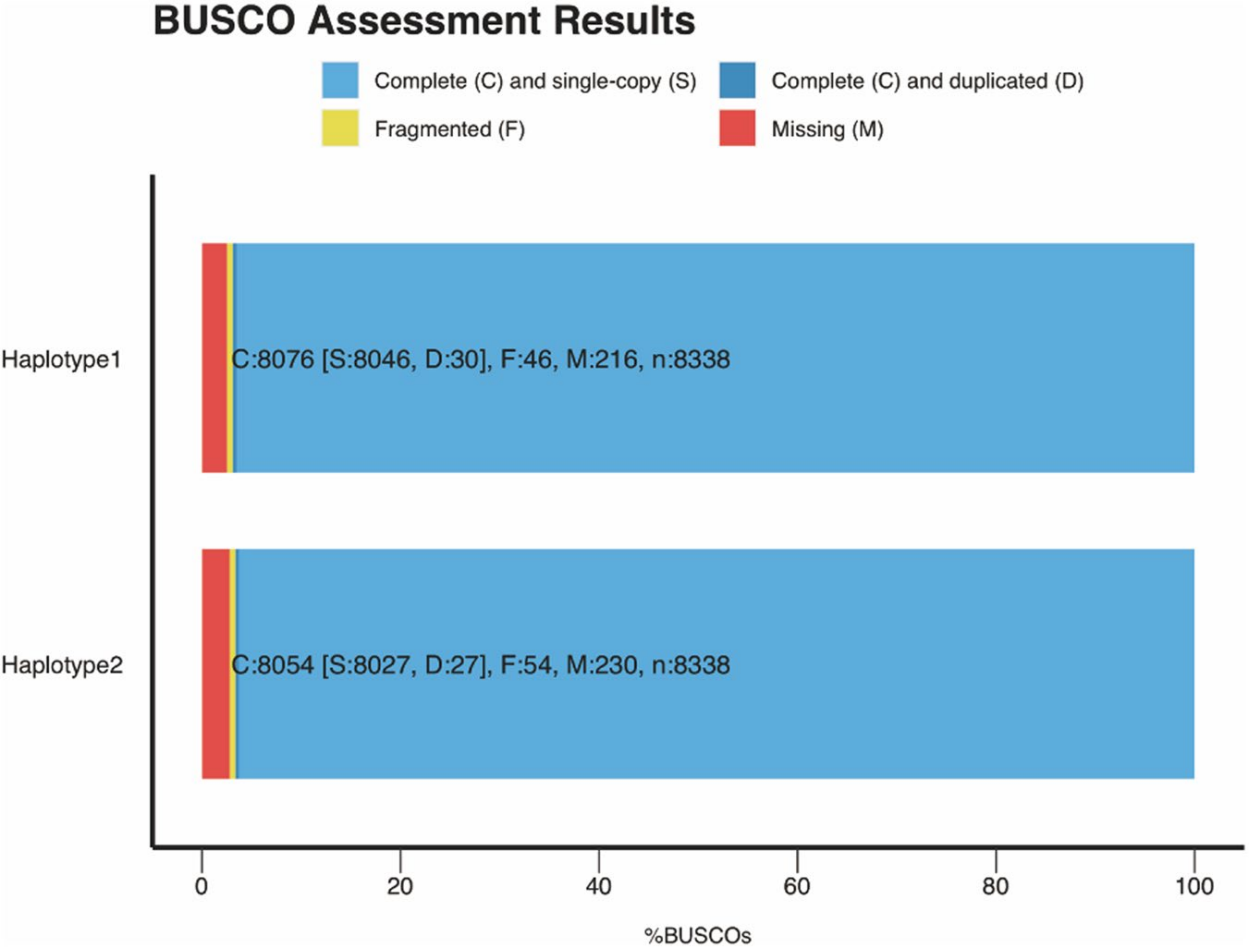
Histogram of BUSCO assessment of the chukar haplotype genome.

We evaluated the completeness of two haplotype genomes via BUSCO v5.1.2^20^ benchmarking using the aves_odb10 dataset. BUSCO reported 96.8% and 96.5% complete universal single-copy orthologs and a low rate of duplication (0.3% and 0.5%) for the Hap1 scaffolds and Hap2 scaffolds, respectively (Fig. 3). This indicated that the genome quality of Hap1 scaffolds and Hap2 scaffolds was better than those of other recently published bird genome assembly^21–24^. Because of the higher completeness and quality of the scaffolded Hap1 assembly, it was selected as the chukar reference genome for downstream analysis. The pairwise genome alignments of Hap1 and Hap2 were performed using MUMer v4.0.0^25^. The collinearity analysis of the Hap1 scaffolds and Hap2 scaffolds is shown in Fig. 2b.

**Fig. 4:**
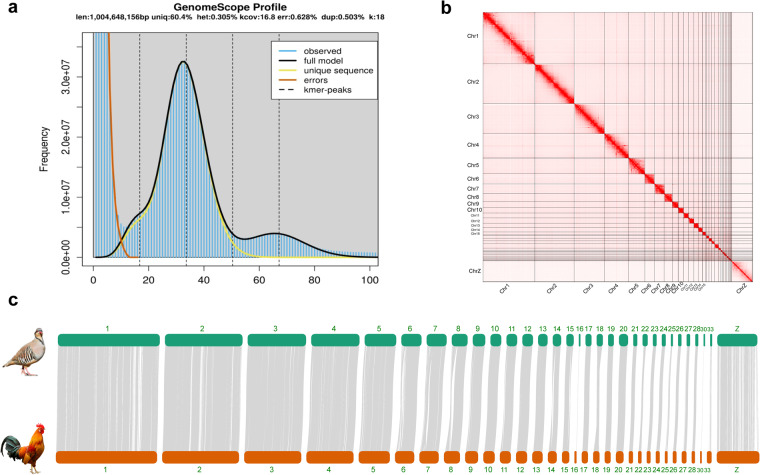
(**a**) The distribution of the k-mer frequency of the chukar genome. (**b**) Genome-wide Hi-C heatmap of *Alectoris chukar*, where both the horizontal and vertical axes represent genomic loci along chromosomes. The chromosomes are arranged sequentially from top to bottom (on the vertical axis) and from left to right (on the horizontal axis), displaying chromosomes Chr1 through Chr28, followed by Chr30, Chr31, Chr33, ChrZ, and ChrW. (**c**)The pairwise genome alignments of the chukar genome and the chicken genome are shown, displaying chromosomes with a length greater than 1 Mb.

### Repeat sequence and gene annotation

To identify genomic repeats, we used the RepeatMasker v4.1.2 (http://www.repeatmasker.org/) to scan the chukar genome sequence of primary assembly. RMBlast v2.11.0 (https://www.repeatmasker.org/rmblast/) was used as the alignment engine. The Dfam 3.0^26^ and Repbase^27^ were used and we specified the species library as ‘chicken’ for the chukar. The overall GC content of the chukar genome was estimated to be 41.81%, which is similar to that of the other reference bird species. Interspersed repeats accounted for approximately 9.8% of the whole genome, spanning 101.47 Mb, and consisted of approximately 90.78 Mb retroelements and 10.27 Mb DNA transposons. Approximately 7.19% of the sequences were identified as long interspersed nuclear elements (LINEs), which were thus the largest component, whereas 1.52% of the sequences were identified as long terminal repeats (LTRs). The chicken repeat 1 group was the most abundant, occupying 99.9% of the identified LINEs. The overall level of repetitive content in the chukar (Table 3) was similar to that in the common pheasant^21^ and chicken^18^ and greater than those of the turkey^7^ as well as most sequenced birds^28^, which may be attributed to the advantage of the long-read sequencing technology.

**Table 3:** The abundance of repeat elements in chukar genome.

Repeat			Count	Length(bp)	Percentage (%)
Retroelements			236,084	90,783,279	8.85%
	SINEs:		4,833	628,711	0.04%
		Penelope	108	22,583	0.00%
	LINEs:		199,242	74,405,324	7.19%
		CRE/SLACS	0	0	0.00%
		CR1/ L2/Rex	199,069	74,367,486	7.19%
		R1/LOA/Jockey	0	0	0.00%
		R2/R4/NeSL	0	0	0.00%
		RTE/Bov-B	0	0	0.00%
		L1/CIN4	65	15,255	0.00%
	LTR elements		32,009	15,749,244	1.52%
		BEL/Pao	0	0	0.00%
		Ty1/Copia	0	0	0.00%
		Gypsy/DIRS1	25	2,812	0.00%
		Retroviral	31,846	15,720,836	1.52%
		ERVL	884	308,637	0.03%
DNA transposons			33,322	10,272,441	0.99%
	hobo-Activator		14,533	5,380,773	0.52%
	Tc1-IS630-Pogo		7,155	3,154,165	0.30%
	PiggyBac		0	0	0.00%
	Tourist/Harbinger		3,845	376,722	0.04%
	Other (Mirage, P-element, Transib)		0	0	0.00%
Rolling-circles			68	9,861	0.00%
Unclassified:			2,492	416,777	0.40%
Total interspersed repeats				101,472,497	9.80%

Gene annotation was performed using a combined strategy of ab initio predictions, homologue prediction, and transcriptome evidence. RNA from the liver, spleen, muscle, thymus gland, bursa of fabricius, and kidney of these two chukars was isolated, library prepared, and sequenced using Illumina technology in Beijing Nuohezhiyuan Technology Service Co, Ltd. Equal amounts of RNA from each of these six tissues were mixed for single-molecule long-read RNA sequencing (Iso-Seq) to obtain full-length transcriptomic data. Illumina reads were filtered by Trimmomatic v0.39^29^ and then combined to input to Trinity v2.14.0 (https://github.com/trinityrnaseq/trinityrnaseq) for transcript assembly. Iso-Seq reads were processed using SMRT tools v5.1.0 and ISO-SEQ v3.1 software packages. The pipeline includes five main steps to obtain high-quality sequences: (1) generating circular consensus (CCS) reads, (2) demultiplexing and primer removal and classifying full-length CCS reads, (3) clustering full-length non-chimeric (FLNC) sequences, and, finally, (4) polishing FLNC sequences (Table S1d). We use PASA v2.5.2 (https://github.com/PASApipeline/PASApipeline) to align assembled transcripts obtained from Illumina reads and Iso-Seq reads to the chukar genome sequences and then TransDecoder v5.50 (https://github.com/TransDecoder/TransDecoder) from the Trinity v2.14.0 to identify the likely open reading frame within the transcripts. The ab initio gene prediction was performed using Augustus based on chicken models^30^. For homology-based annotation, GeMoMa v1.9^31^ was employed using gene annotation information from chicken. Finally, all the results were integrated using the EVidenceModeler pipeline v1.1.1^32^. Alternative splicing analysis of the transcripts uses SUPPA v2.3^33^, which generates seven different alternative splicing types, including skipped exon (SE), alternative 5′/3′ splice sites (A5/A3), mutually exclusive exons (MX), retained intron (RI) and alternative first/last exons (AF/AL). The final gene models comprised 20,082 transcripts, spanning a 302 Mb genomic region. These genes were annotated by using the UniProt/SwissProt protein database and validated 17,997 protein products. In addition, 109,477 splicing events were identified corresponding to all genes. Among seven type splicing events, the largest number of splicing events in chukar was alternative first exons (28,722, 26%), followed by Retained Intron (25865, 24%) and alternative 5′ splice site (17235, 16%) (Table S2b).

### Population-based resequencing and variation calling

Genomic resequencing was performed for each individual on MGI-SEQ. 2000 platform. Raw reads were subjected to SOAPnuke v1.5.0^12^ processing to remove sequencing adapters and low-quality reads. Following whole-genome resequencing of domestic chukar and five wild chukar subspecies, a total of 1,071.8 Gb of the clean base was obtained, with an average depth of 18× (Table S1b). High-quality reads were aligned to the chukar genome that we assembled above using the Burrows−Wheeler Aligner v0.5.9^34^. Variants were called using the GATK tool suite v4.2.6.1^35^. Briefly, potential PCR duplicates were marked using MarkDuplicates option. The HaplotypeCaller option was used to construct general variant calling files for all the samples by invoking -ERC:GVCF. All of gVCF files were combined using GenotypeGVCFs option to form a single variant calling file. To obtain high-quality SNPs and Indels, we used the GATK hard filter to filter the merged VCF data with the best practices recommended parameters^36^. After quality filtering, 2,574,885 high-quality INDELs and 14,988,840 high-quality SNPs were identified. Following the removal of SNPs and INDELs using vcftools v0.1.16^37^ with the parameters ‘–mac 3–maf 0.05–min-meanDP 5–minQ 30–max-missing 0.95’, a total of 6,991,669 SNPs and 757,682 INDELs from 58 individuals were retained.

## Data Records

The chukar genome assembly reported in this paper have been deposited in the Genbank under the project PRJNA780965 with the accession number JAXHPU000000000^38^. The variation files for this study are located under analysis ERZ22149693 at EVA (European Variation Archive), with the accession number PRJEB7133960^39^. The PacBio sequencing data (SRR27640724) were specifically used to construct the genome assembly. For variant analysis, we utilized whole-genome resequencing data (SRR16961228-SRR16961264). Additionally, transcriptome data from (SRR26796665-SRR26796677 and SRR27640723) informed our genome annotation. These datasets, integral to our project, are available through NCBI BioProject PRJNA780965^40^.

## Technical Validation

Jellyfish was used in conjunction with GenomeScope^14^ to calculate genome size and heterozygosity in the chukar genome using a k-mer frequency of 18. The genome was consistent with the genome size estimated (1.0 Gb) (Fig. 3a). The Hi-C heatmap revealed a well-organized interaction contact pattern along the diagonals within/around the chromosome (Fig. 3b), which indirectly confirmed the accuracy of the chromosome assembly. The pairwise genome alignments of the chukar genome and the chicken genome using NGenomeSyn^41^ demonstrate a high level of consistency, which also implies that the genome assembly is accurate and reliable (Fig. 3c). To further assess the quality of assembled chukar genome, we have undertaken an extensive quality assessment by mapping 58 genome resequencing samples to it. The resulting quality metrics, including a coverage of over 99%, a sequencing depth greater than 15X, and a mapping rate of more than 98%, are detailed in Table S3. These metrics not only confirm the technical soundness but also the high quality of the genome assembly. We also evaluated the completeness of two haplotype genomes via BUSCO v5.1.2 benchmarking using the aves_odb10 dataset^20^. BUSCO reported 96.8% and 96.5% complete universal single-copy orthologs and a low rate of duplication (0.3% and 0.5%) for the Hap1 scaffolds and Hap2 scaffolds, respectively (Fig. 3).

### Supplementary information

Table S1

Table S2

Table S3

## Data Availability

The genome and transcriptome analyses were performed following the manuals and protocols of the cited bioinformatic sofware. No new codes were written for this study.
